# First record of the little-known family Katianiridae Svavarsson, 1987 (Crustacea, Isopoda, Asellota) from the Southwest Atlantic Ocean, with description of two new species

**DOI:** 10.3897/zookeys.1295.205971

**Published:** 2026-10-20

**Authors:** Sofía Calderón López, Daniel Roccatagliata, Brenda Doti

**Affiliations:** 1 Universidad de Buenos Aires, Facultad de Ciencias Exactas y Naturales, Departamento de Biodiversidad y Biología Experimental (DBBE), Buenos Aires, Argentina Universidad de Buenos Aires, Facultad de Ciencias Exactas y Naturales, Departamento de Biodiversidad y Biología Experimental (DBBE) Buenos Aires Argentina; 2 Universidad de Buenos Aires – CONICET, Instituto de Biodiversidad y Biología Experimental y Aplicada (IBBEA), Ciudad Universitaria, Buenos Aires, Argentina Universidad de Buenos Aires – CONICET, Instituto de Biodiversidad y Biología Experimental y Aplicada (IBBEA) Buenos Aires Argentina

**Keywords:** Argentina, deep-sea, identification key, *
Natalianira
*, new records, new species, range extension, taxonomy

## Abstract

For the first time, species of Katianiridae are recorded from the Southwest Atlantic Ocean: *Natalianira bicornuta***sp. nov**. from the Mar del Plata submarine canyon and *Natalianira megalocera***sp. nov**. from the Marine Protected Area Namuncurá-Burdwood Bank are described. Both species can be distinguished from *Natalianira spinosa* Kensley, 1984, the only currently known species of this genus, by having (1) distal margin of the first article of the antennula produced, (2) a single row of mid-dorsal bumps on the pereonites 1–7, and (3) uropods distinctly extending beyond the posterior margin of the pleotelson. The geographic and bathymetric ranges of the genus *Natalianira* are extended.

## Introduction

Svavarsson (1987) reevaluated the taxonomic placement of the genera *Katianira* Hansen, 1916 and *Natalianira* Kensley, 1984, and erected the family Katianiridae to accommodate both genera. This family currently includes six species, i.e., *Katianira* (5 spp.) and *Natalianira* (1 sp.).

With the exception of *Katianira setifera* (Kussakin, 1982), which was reported from Antarctica, the remaining four species of the genus *Katianira* are known from the northern hemisphere, namely, *K. chelifera* Hansen, 1916 (type species) and *K. acarina* (Menzies, 1962) both from the North Atlantic; *K. bilobata* Gurjanova, 1930 from the North Polar Sea; and *K. platyura* Shimomura & Akiyama, 2006 from the North Pacific. In addition, Wolff (1975) reported an undescribed species of this genus from the Caiman Trench (Yucatan Basin, Caribbean Sea). In contrast, the genus *Natalianira* includes only one species, *N. spinosa* Kensley, 1984, from South Africa.

The genus *Katianira* has been recorded over a wide bathymetric range, from sublittoral to abyssal depths (5–5779 m) (Hansen 1916; Gurjanova 1930, 1933, 1946, 1964; Menzies 1962; Wolff 1962, 1975, 1979; Kussakin 1982, 1988; Svavarsson 1987; Shimomura and Akiyama 2006). Conversely, the monotypic genus *Natalianira* is known only from the bathyal zone, in a range of 550–850 m depth (Kensley 1984).

In the current study, two new species of the genus *Natalianira*, both collected on the continental slope off the coast of Argentina, are described: *N. bicornuta* sp. nov. from the Mar del Plata submarine canyon and *N. megalocera* sp. nov. from the Marine Protected Area Namuncurá-Burdwood Bank and off Isla de los Estados. The diagnosis of this genus is emended. Moreover, dichotomous keys to the current genera and the three species of *Natalianira* are also provided.

## Material and methods

The specimens were collected using an epibenthic sledge similar to the one designed by Hessler and Sanders (1967), equipped with a net of 1 mm mesh size, during the expeditions “Talud Continental I and II” (2012 and 2013, respectively) and “Bentos Banco Burdwood” (2016). On board the RV Puerto Deseado, the samples were sieved with a 300 µm mesh size. Part of the material collected was fixed with 10% seawater-buffered formalin on board and transferred to 70% ethanol in the laboratory. Another part was preserved directly in 96% ethanol.

The specimens described were stained with Chlorazole Black E® and the appendages were dissected and temporarily mounted in glycerin. Habitus drawings were made using a camera lucida attached to a Leica MZ8 dissecting microscope. Appendages were drawn using a Carl Zeiss (Axioskop) compound microscope also equipped with a camera lucida. Line drawings were rendered in a digital format using a Wacom tablet and Adobe Illustrator.

For SEM photographs, the specimens were cleaned with 0.5% nonionic detergent TWEEN 20 and ultrasound. The specimens were dehydrated through a graded series of ethanol ending in 100%. Afterwards, the specimens were transferred to increasing concentrations of hexamethyldisilazane (HMDS). Specimens in 100% HMDS were allowed to air-dry and then mounted on aluminum stubs, gold-palladium sputter-coated, and examined under a Philips XL30 TMP microscope. Body lengths were measured in dorsal view from the frontal margin of the head to the tip of the pleotelson along the midline. Appendage measurements were taken following Hessler (1970).

The type and additional material of *Natalianira bicornuta* sp. nov. and *N. megalocera* sp. nov. were deposited in the invertebrate collection of the Museo Argentino de Ciencias Naturales “Bernardino Rivadavia”.

Abbreviations: MdPSC, Mar del Plata submarine canyon; MPA N-BB, Marine Protected Area Namuncurá-Burdwood Bank; MACN, Museo Argentino de Ciencias Naturales “Bernardino Rivadavia”; BBB, Bentos Banco Burdwood expedition; Talud I and II, Talud Continental I and II expeditions.

## Results

### Taxonomy

**Katianiridae Svavarsson, 1987**

#### Natalianira

Taxon classification

Animalia

IsopodaKatianiridae

Kensley, 1984

F814C1C9-8C72-5A54-B286-FE73BA83E147

Natalianira Kensley, 1984: 279; Svavarsson 1987: 719.

##### Type species.

*Natalianira spinosa* Kensley, 1984 (by monotypy).

##### Species included.

*Natalianira spinosa* Kensley, 1984; *N. bicornuta* sp. nov.; *N. megalocera* sp. nov.

##### Diagnosis

**(modified from Kensley 1984; Svavarsson 1987)**. Body, lateral margins with robust setae, mid-dorsal surface with bumps. Pleotelson with lateral lobes or processes. Antennula article 1 greatly enlarged with robust setae marginally. Antenna flagellum short with 1 or 2 articles. Mandible, setal row reduced, molar process spiniform. Maxillipedal palp with 4 (5) articles. Pereopod 1 with chela, formed between the dactylus claw and the thick ventrodistal seta of the propodus; dactylus short and wide proximally. Uropods flat or cylindrical with numerous robust setae marginally.

#### Natalianira bicornuta sp. nov.

Taxon classification

Animalia

IsopodaKatianiridae

E5563640-DE9F-586B-9FC6-074931428436

https://zoobank.org/ADAE22B9-5273-4F97-9F65-8B34BEC208A3

Figs 1, 2, 3, 4

##### Material examined.

***Holotype***: Argentina • ♂ (2.8 mm); Mar del Plata submarine canyon; Sta. 12, 37°57.91'S, 54°31.92'W; 1144 m; 11 Aug. 2012; Talud I; RV Puerto Deseado; Coll. I. Chiesa; MACN-In 45613. ***Paratypes***: Argentina • 8 ♂♂ (2.1–2.8 mm), 1 ovigerous ♀ (2.2 mm), 4 juveniles (1.2–1.8 mm), 3 mancas (0.8–0.9 mm); same data as holotype; MACN-In 45614.

**Figure 1.**
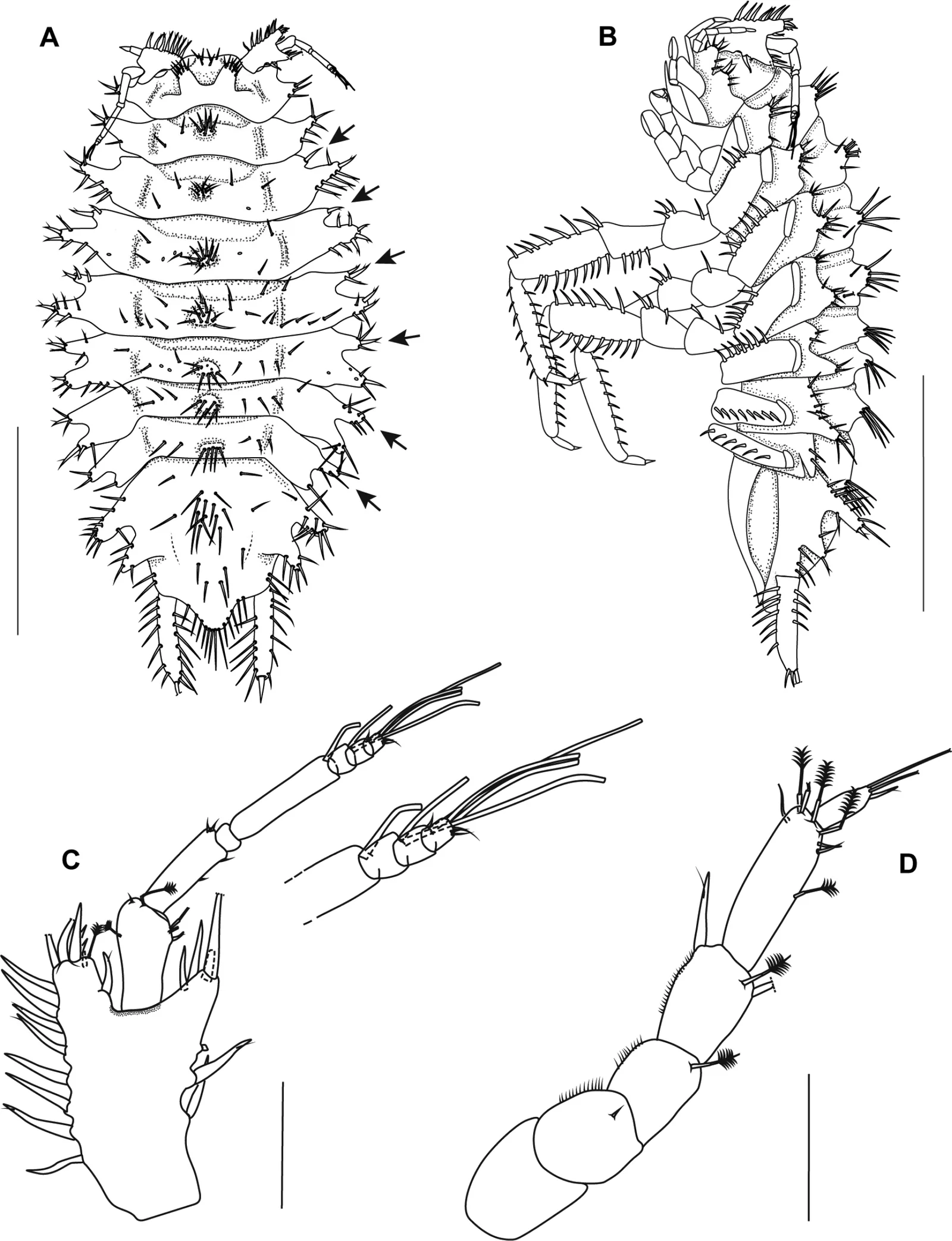
*Natalianira bicornuta* sp. nov. Holotype male (MACN-In 45613). **A, B**. Habitus in dorsal and lateral views, respectively; arrows point to the anterior lobe on pereonites 2–7. Paratype male (MACN-In 45614-a); **C**. Antennula, with detail of the distal articles; **D**. Antenna. Scale bars: 1 mm (**A, B**); 0.1 mm (**C, D**).

**Figure 2.**
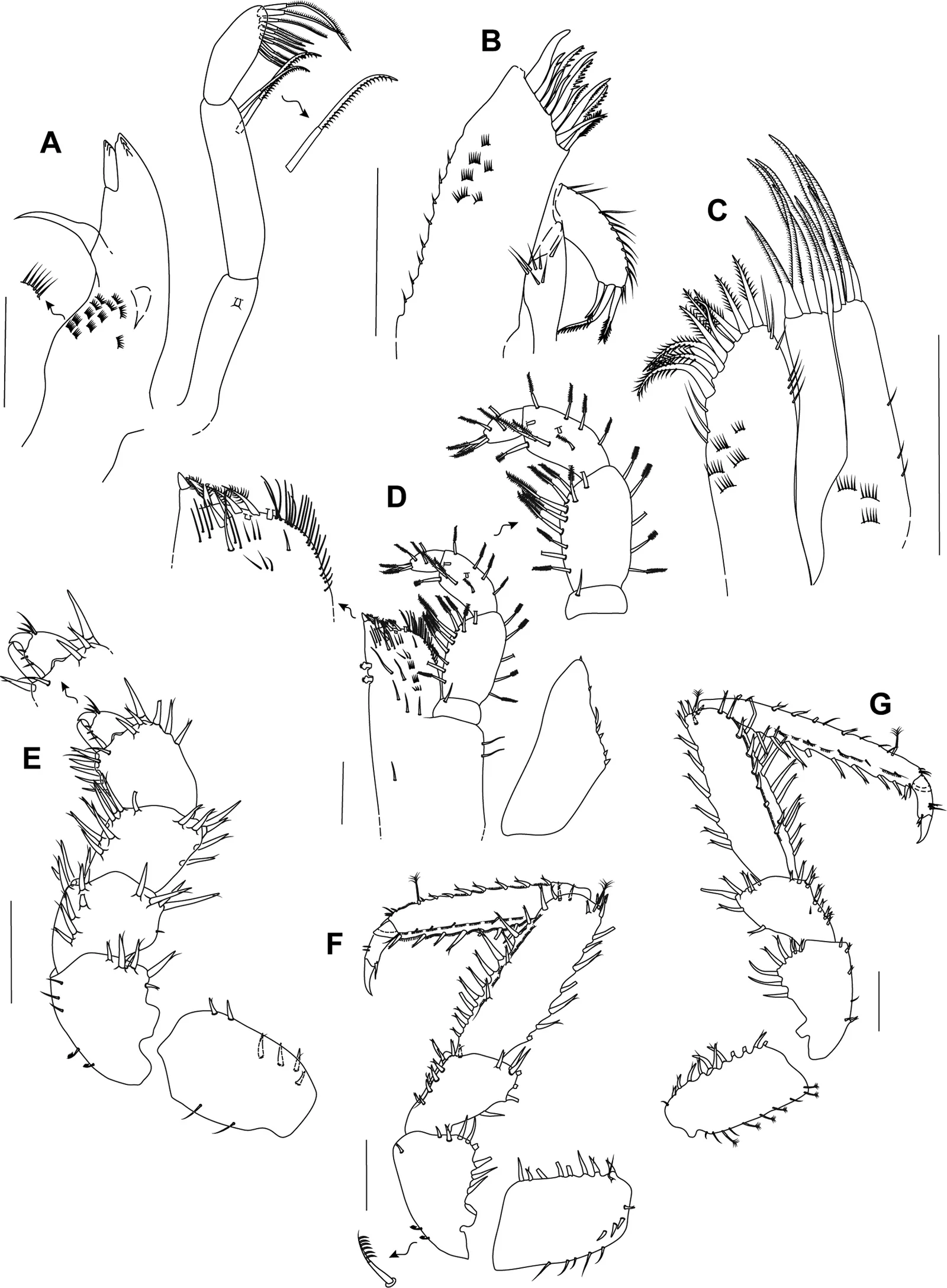
*Natalianira bicornuta* sp. nov. Paratype male (MACN-In 45614-a). **A**. Left mandible; **B**. Maxilulla; **C**. Maxilla; **D**. Maxilliped, with detail of distal margin of endite and detail of the maxillipedal palp; **E–G**. Pereopods 1, 2 and 7, respectively, with detail of pereopod 1 chela. Scale bars: 0.1 mm (**A–D**); 0.2 mm (**E–G**).

**Figure 3.**
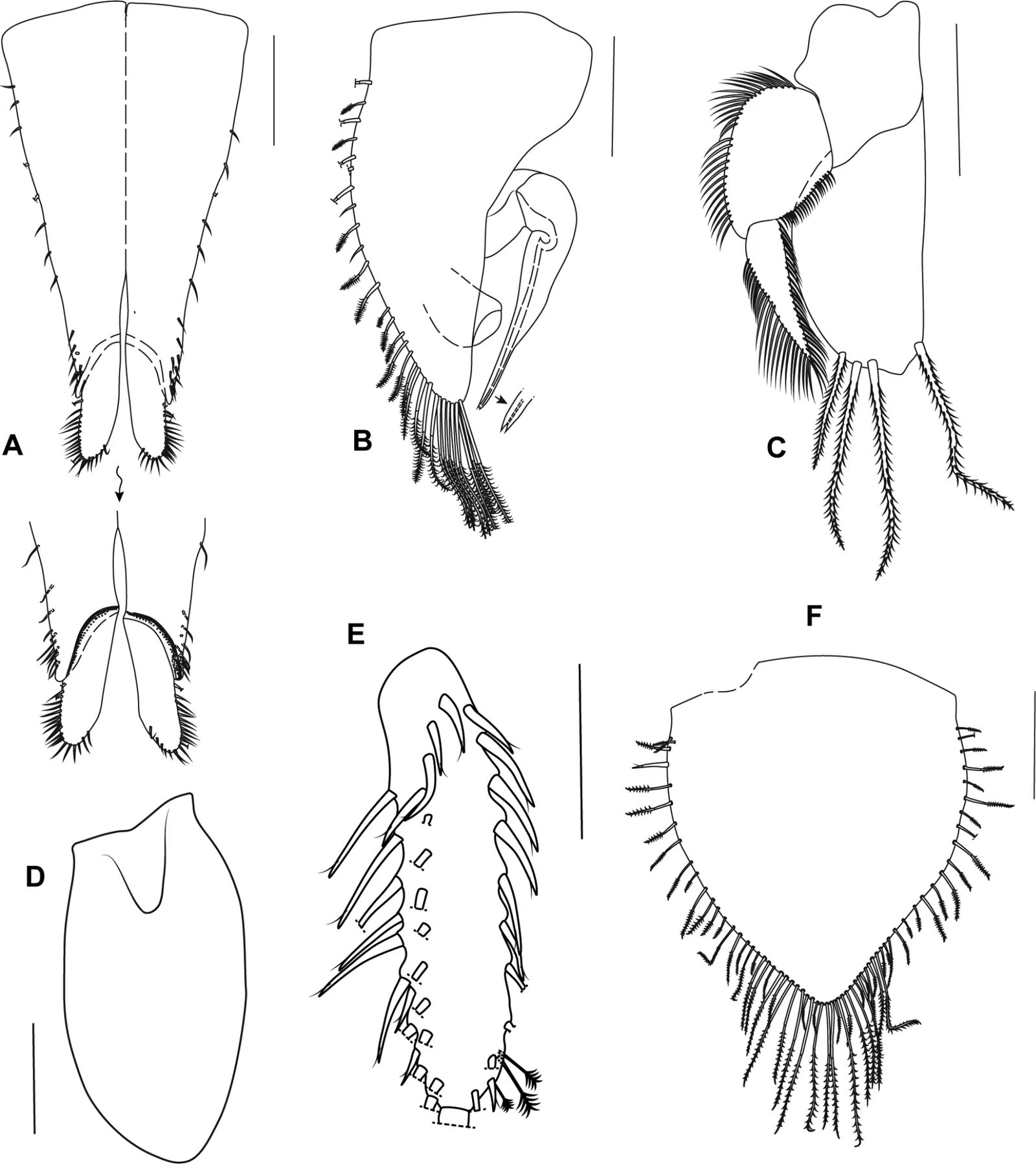
*Natalianira bicornuta* sp. nov. Paratype male (MACN-In 45614-a). **A–D**. Pleopods 1–4, respectively, with detail of pleopod 1 distal lobes; **E**. Uropod. Paratype female (MACN-In 45614-b); **F**. Operculum. Scale bars: 0.2 mm (**A–C, E, F**); 0.1 mm (**D**).

**Figure 4.**
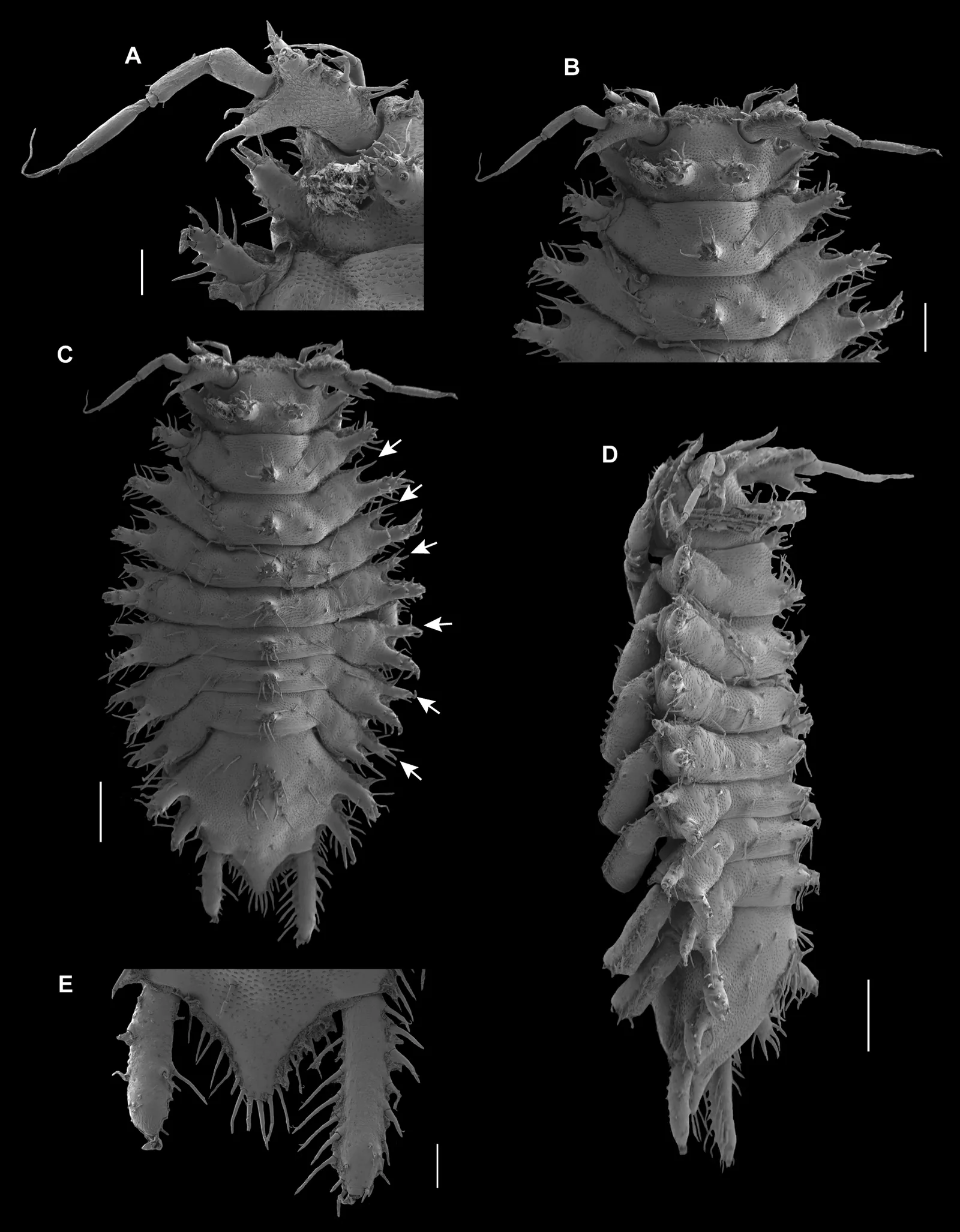
*Natalianira bicornuta* sp. nov. Paratype male (MACN-In 45614-c). **A**. Antennula; **B**. Head and first two pereonites; **C, D**. Habitus in dorsal and lateral views, respectively; arrows point to the anterior lobe on pereonites 2–7; **E**. Detail of posterior margin of pleotelson and uropods. Scale bars: 0.1 mm (**A, E**); 0.2 mm (**B**); 0.3 mm (**C, D**).

##### Additional material.

Argentina • Mar del Plata submarine canyon, Talud II, RV Puerto Deseado, Colls. I. Chiesa and A. Martinez: 4 specimens; Sta. 39, 37°58.05'S, 54°31.72'W; 1144 m; 25 May 2013; MACN-In 45615 • 18 specimens; Sta. 37, 37°59.85'S, 54°24.21'W; 1275 m; 25 May 2013; MACN-In 45616.

##### Description of adult male

**(Habitus based on holotype MACN-In 45613, appendages based on paratype MACN-In 45614-a)**. Body (Figs 1A, 1B, 4C, 4D) length 1.8 at greatest width, widest at pereonite 4. Head anterior margin with robust setae (Figs 1A, 4B); lateral margin produced; dorsal surface with two large cylindrical protuberances, each protuberance with 8–10 robust setae apically. All pereonites with one mid-dorsal bump with robust setae apically and some additional setae on either side of the mid-dorsal bumps; lateral margins with robust setae. Pereonites 1–4 gradually increasing in width; pereonites 5–7 gradually decreasing in width. Lateral margin of pereonite 1 produced, lateral margins of pereonites 2–7 bilobed; anterior lobe gradually increasing in length from pereonites 2 to 5, becoming as long as posterior lobe in pereonite 6, and longer than posterior lobe on pereonite 7. Pleotelson, lateral margin with two large processes, each process with 8–10 robust setae; dorsal surface with robust setae, mainly on the midline.

Antennula (Figs 1C, 4A) peduncle article 1 length 1.8 width, with 1 medial and 1 lateral processes subequal in length, both reaching half-length of article 2, medial margin with 15 robust setae and 1 broom seta, lateral margin with 7 robust setae; article 2, length 0.4 article 1 length, margins with 4 broom setae (2 broken), 1 simple seta and 1 robust seta; article 3 length 0.4 article 1 length, margins with 4 simple setae. Flagellum 5-articled, 0.8 peduncle article 1 length; article 1 ring-like, length 0.1 article 2 length, glabrous; article 2 longest; articles 2–5 with 1–2 aesthetascs each; articles 4–5 with 1–3 additional simple setae.

Antenna (Fig. 1D) peduncle 5-articled; article 1 length 1.4 width, glabrous; articles 2–4 with small simple setae marginally; article 2 length 0.7 article 5 length with 1 simple seta; article 3 length 0.6 article 5 length with 1 broom seta; article 4 length 0.7 article 5 length with 2 robust setae and 1 broom seta; article 5 longest article, length 3.0 width, with 3 simple and 5 broom setae. Flagellum 1-articled, distal margin with 1 robust and 2 simple setae.

Mandibles, palp 3-articled, incisor process denticulate, molar process spiniform. Left mandible (Fig. 2A) with rounded condyle, *lacinia mobilis* 4-denticulate; palp, article 1 with 1 pectinate seta distally (broken, observed in right mandible), article 2 with 2 pectinate setae distally; article 3 with 10 pectinate setae distally.

Maxillula (Fig. 2B) medial endite (broken) with 3 setulate setae distally, lateral margin and ventral surface with several simple setae; lateral endite distal margin with 1 plumose, 3 denticulate, 2 setulate and 6 cuspidate setae (largest broken, observed on the right maxillula), surface with several cuticular combs, lateral margin with 8 simple setae.

Maxilla (Fig. 2C) medial endite with 9 setulate and 6 simple setae distally; margins with simple setae; both lobes of lateral endite with 4 serrate setae distally; ventral surface with cuticular combs (only a few drawn).

Maxilliped (Fig. 2D) endite, medial margin with 2 coupling hooks, distal margin with 5 robust and 4 setulose setae, lateral margin with several simple setae, ventral surface with some large and short simple setae and cuticular combs. Palp 4-articled; article 1 with 1 simple seta distally; article 2 with 10 setulate setae medially and 5 setulate setae laterally; article 3 with 10 setulate setae (2 broken); article 4 with 5 setulate setae and 1 robust seta.

Pereopod 1 (Fig. 2E) shortest and robust, basis longest article, articles length ratio (as the relative length of each article to propodus): 2.0: 1.3: 0.8: 0.8: 1.0: 0.4. Basis ventral margin with 2 simple setae; dorsal margin with 6 robust setae. Ischium ventral margin with 3 simple and 2 small pectinate setae; dorsal margin with 6 robust setae. Merus ventral margin with 9 robust setae; dorsal margin with 4 robust setae and 1 simple seta. Carpus ventral margin with 8 robust setae; dorsal margin with 6 robust setae. Propodus ventral margin with 6 robust and 2 simple setae; distal-most robust seta largest and opposed to dactylus claw forming the chela; dorsal margin with 6 robust setae. Dactylus with 1 distal claw; ventral margin with 3 simple setae; dorsal margin with 3 simple setae.

Pereopods 2–7 gradually increasing in length.

Pereopod 2 (Fig. 2F) propodus longest article, article length ratio (as the relative length of each article to propodus): 0.6: 0.5: 0.3: 0.7: 1.0: 0.3. Basis ventral margin with 5 simple and 4 robust setae; dorsal margin with 1 simple and 8 robust setae. Ischium ventral margin with 1 robust and 2 small pectinate setae; dorsal margin with 8 robust setae. Merus ventral margin with 10 robust setae; dorsal margin with 7 robust setae. Carpus ventral margin with 17 robust setae; dorsal margin with 1 broom and 12 robust setae. Propodus ventral margin with 8 robust and 5 simple setae; dorsal margin with 8 robust setae, 4 simple setae and 1 broom seta. Dactylus distal margin with 1 large claw; ventral margin with 1 robust seta and 1 simple seta; dorsal margin with 2 simple setae.

Pereopod 7 (Fig. 2G) propodus longest article, article length ratio (as the relative length of each article to propodus): 0.6: 0.6: 0.5: 0.9: 1.0: 0.4. Basis ventral margin with 4 simple and 6 broom setae; dorsal margin with 1 simple and 11 robust setae. Ischium ventral margin with 1 simple and 5 robust setae; dorsal margin with 8 robust setae. Merus ventral margin with 10 robust setae and 1 simple seta; dorsal margin with 7 robust setae. Carpus ventral margin with 19 robust setae; dorsal margin with 1 broom and 11 robust setae. Propodus ventral margin with 9 robust and 6 simple setae; dorsal margin with 5 robust setae, 8 simple setae and 1 broom seta. Dactylus distal margin with 1 large claw; ventral margin with 1 robust seta and 1 simple seta; dorsal margin with 4 simple setae.

Pleopod 1 (Fig. 3A) length 1.8 at greatest width, with two distal lobes outlined by two furrows; lateral margins with 15–18 simple setae; lobes distally rounded marginally setulate.

Pleopod 2 (Fig. 3B) protopod length 1.8 at greatest width, lateral margin with 2 simple and 28 setulate setae (some broken); endopod with two articles, article 2 stylet-form with a distal pore (see detail) reaching distal tip of protopod.

Pleopod 3 (Fig. 3C) protopod glabrous; endopod subequal in length to exopod, distal margin with 4 plumose setae; exopod 2-articled with many simple setae marginally.

Pleopod 4 (Fig. 3D) uniramous and glabrous.

Pleopod 5 absent.

Uropod (Figs 3E, 4E) cylindrical, length 4.3 at greatest width, with 35 robust setae, distal-most largest, and 3 broom setae (some setae broken).

##### Description of adult female

**(based on paratype MACN-In 45614-b)**. As male except for:

Operculum (Fig. 3F) subtriangular, length 1.2 at greatest width, narrowing from distal half to the rounded apex; with 1 robust seta on proximo-lateral margin (present only on left side) and 63 setulate setae, longest setae at distal end.

##### Remarks.

*Natalianira bicornuta* sp. nov. can be distinguished from *N. spinosa* Kensley, 1984 and *N. megalocera* sp. nov. by the presence of two large dorsal processes on the head and two pairs of large lateral processes on the pleotelson.

##### Etymology.

The species epithet combines two Latin terms: “bi”, meaning two, and “cornata”, meaning horn. It refers to the two large dorsal horns on the head of this species.

##### Distribution.

This new species is only known from the Mar del Plata submarine canyon, Argentina between 1114–1275 m depth (Fig. 9).

#### Natalianira megalocera sp. nov.

Taxon classification

Animalia

IsopodaKatianiridae

14EC0238-8F6A-5C50-850E-4FD763DF5DB0

https://zoobank.org/407AE319-6179-42FB-812F-CB5C216CD42E

Figs 5, 6, 7, 8

##### Material examined.

***Holotype***: Argentina • ♀ (2.5 mm); off Isla de los Estados; Sta. 13 (L.173), 54°36.24'S, 62°50.91'W; 647 m; 9 Apr. 2016; BBB 2016; RV Puerto Deseado; Colls. I. Chiesa and D. Roccatagliata; MACN-In 45617. ***Paratypes***: Argentina • 1 ♀ (2.3 mm), 7 ♂♂ (1.8–2.0 mm), 1 juvenile (1.7 mm), 6 mancas (1.5–1.6 mm); same data as holotype; MACN-In 45618.

**Figure 5.**
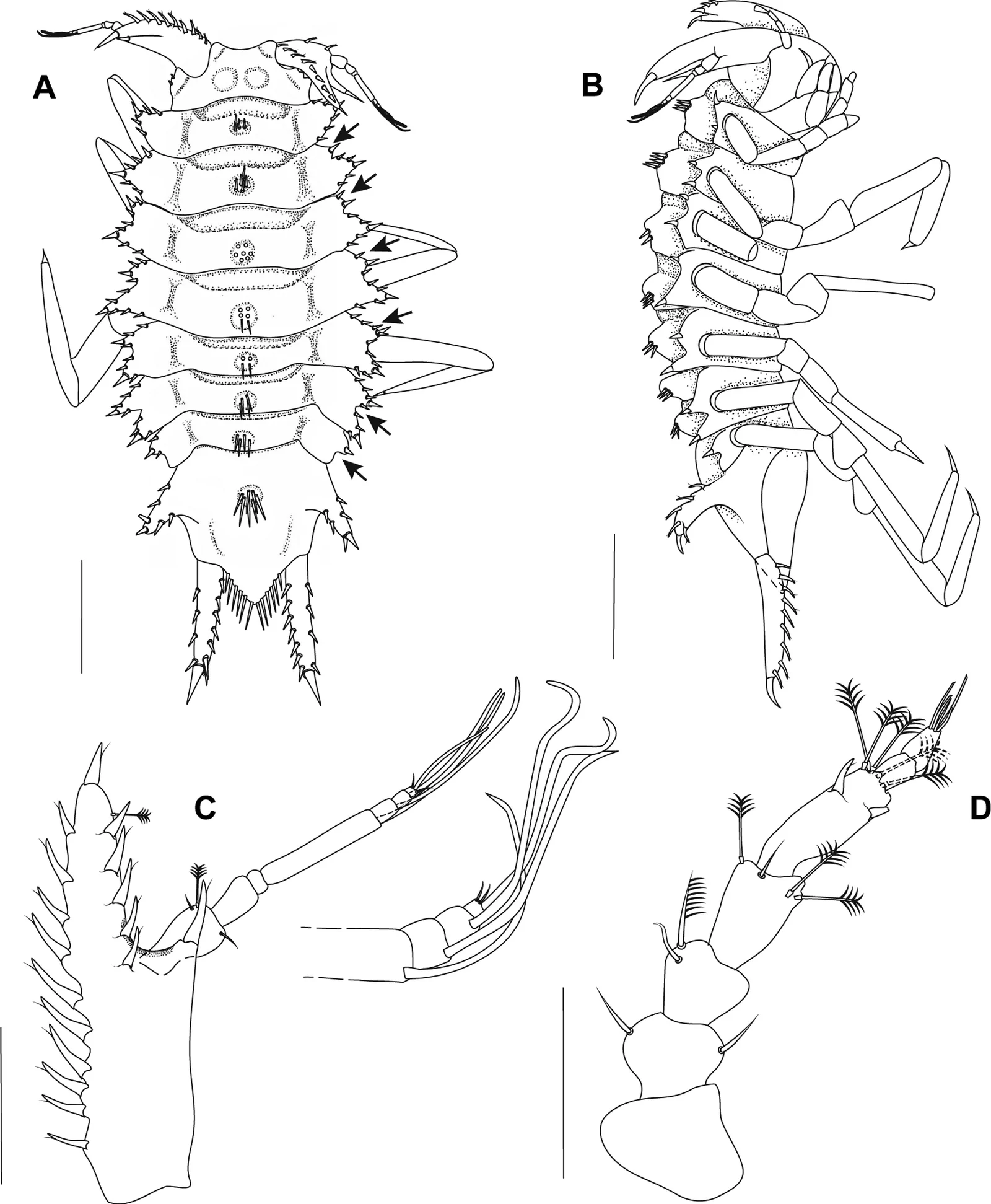
*Natalianira megalocera* sp. nov. Holotype female (MACN-In 45617). **A, B**. Habitus in dorsal and lateral views, respectively; arrows point to the anterior lobe on pereonites 2–7. Paratype female (MACN-In 45618-a); **C**. Antennula, with detail of the distal articles; **D**. Antenna. Scale bars: 0.5 mm (**A, B**); 0.25 mm (**C**); 0.1 mm (**D**).

**Figure 6.**
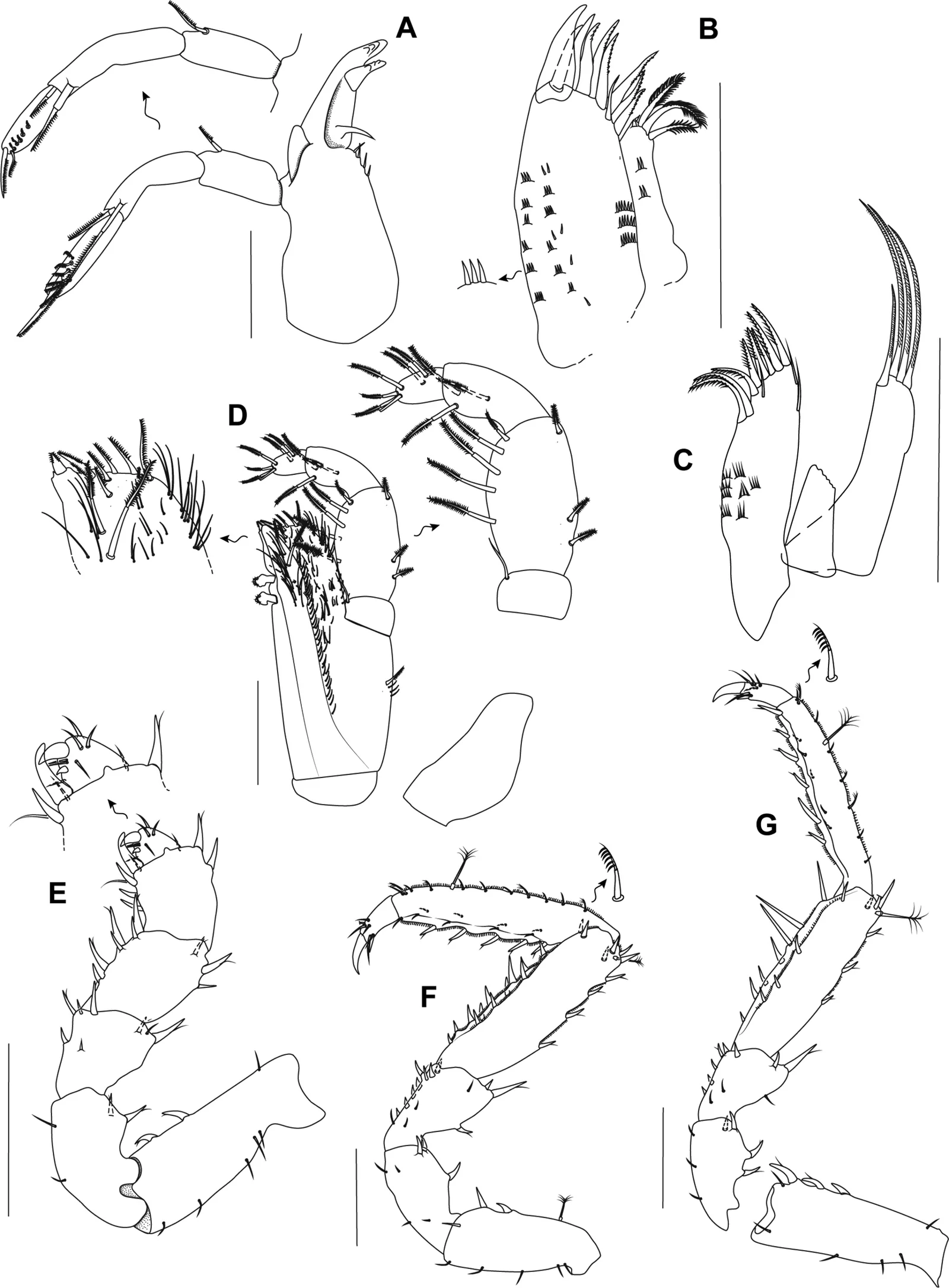
*Natalianira megalocera* sp. nov. Paratype female (MACN-In 45618-a). **A**. Left mandible; **B**. Maxilulla; **C**. Maxilla; **D**. Maxilliped, with detail of distal margin of endite and detail of the maxillipedal palp; **E–G**. Pereopods 1, 2 and 7, respectively, with detail of pereopod 1 chela. Scale bars: 0.1 mm (**A–D**); 0.2 mm (**E–G**).

**Figure 7.**
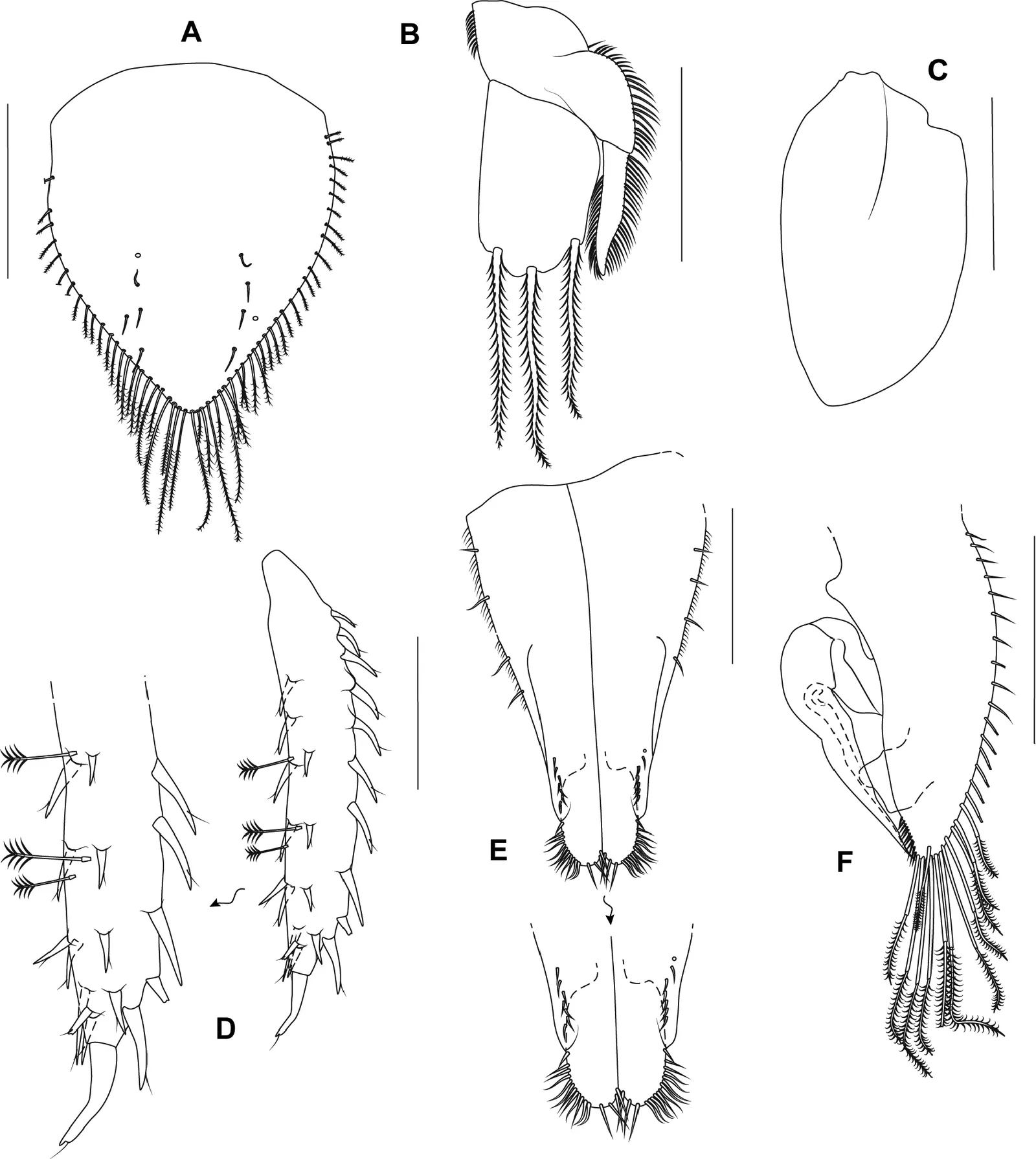
*Natalianira megalocera* sp. nov. Paratype female (MACN-In 45618-a). **A**. Operculum; **B, C**. Pleopods 3 and 4, respectively; **D**. Uropod. Paratype male (MACN-In 45618-b); **E, F**. Pleopods 1 and 2, respectively; with detail of pleopod 1 distal lobe. Scale bars: 0.2 mm (**A, B, D–F**); 0.1 mm (**C**).

**Figure 8.**
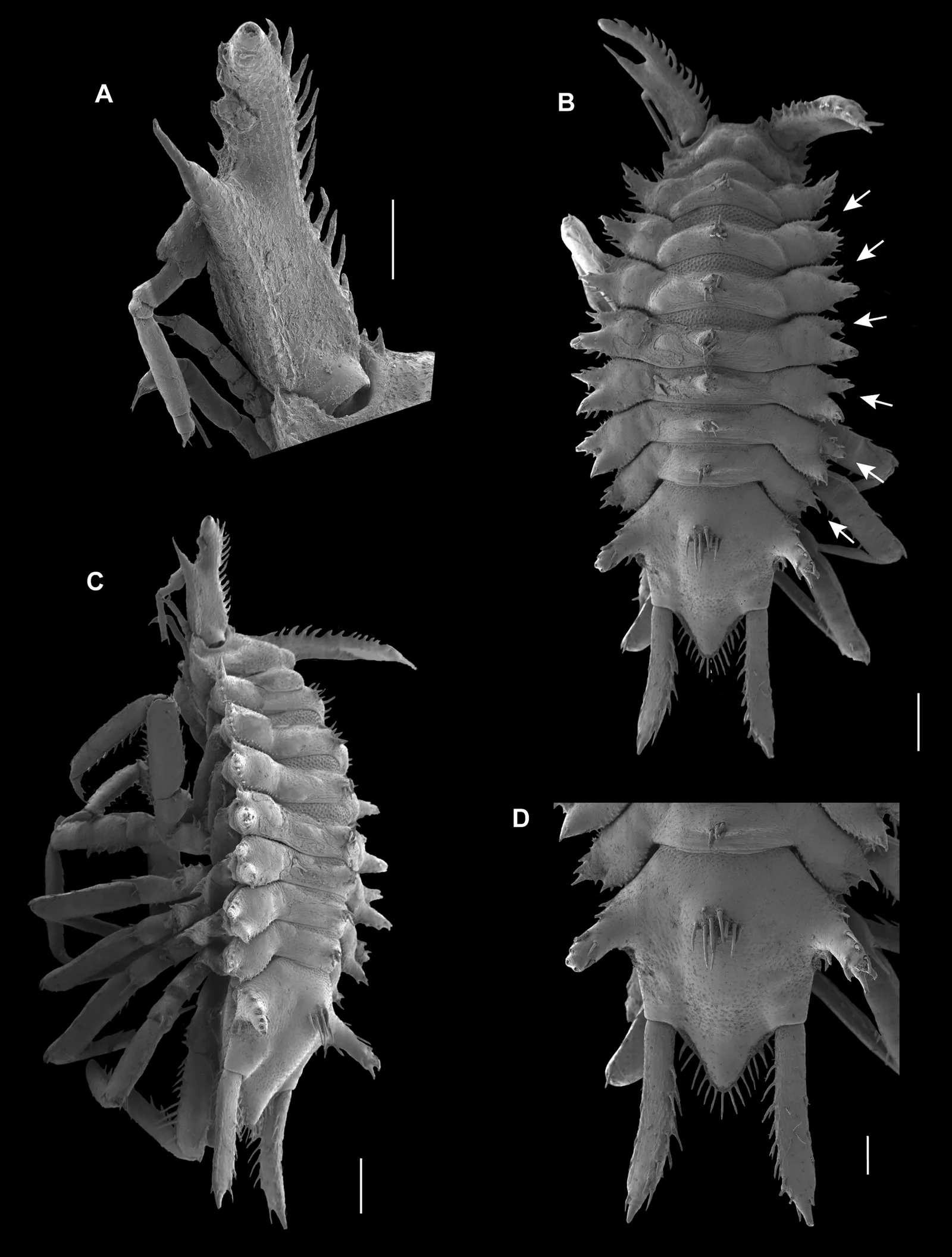
*Natalianira megalocera* sp. nov. Paratype female (MACN-In 45618-c). **A**. Antennula; **B, C**. Habitus in dorsal and lateral views, respectively; arrows point to the anterior lobe on pereonites 2–7; **D**. Detail of pleotelson and uropods. Scale bars: 0.2 mm (**A**); 0.3 mm (**B, C**); 0.1 mm (**D**).

##### Additional material.

Argentina • 16 specimens; Marine Protected Area Namuncurá-Burdwood Bank; Sta. 16 (L. 334), 54°50.20'S, 61°30.82'W; 846 m; 20 Apr. 2016; BBB 2016, RV Puerto Deseado; Colls. I. Chiesa and D. Roccatagliata; MACN-In 45619.

##### Description of adult female

**(Habitus based on holotype MACN-In 45617, appendages based on paratype MACN-In 45618-a)**. Body (Figs 5A, 5B, 8B, 8C) length 1.9 at greatest width, widest at pereonite 4. Head, dorsal surface with two low rounded glabrous bumps. All pereonites with one mid-dorsal low bump with robust setae apically; lateral margins with robust setae. Pereonites 1–4 gradually increasing in width; pereonites 5–7 gradually decreasing in width. Lateral margin of pereonite 1 produced; lateral margins of pereonites 2–7 bilobed; anterior lobe gradually increasing in length from pereonites 2 to 5, becoming as long as posterior lobe in pereonite 6, and longer than posterior lobe on pereonite 7. Pleotelson (Fig. 8D), lateral margin with one enlarged process with 9–11 robust setae; dorsal surface with a group of robust setae at midline.

Antennula (Figs 5C, 8A) peduncle article 1 length 3.2 width, distal margin with two processes: lateral process short, distal margin with 1 robust seta, medial process greatly produced, extending beyond article 4, with 16 robust setae and 1 broom seta; article 2 length 0.2 article 1 length, with 2 simple setae and 1 broom seta; article 3 length 0.1 article 1 length, glabrous; flagellum 5-articled, 0.5 peduncle article 1 length; article 1 ring-like, length 0.1 article 2 length, glabrous; article 2 longest, as long as preceding 3 articles combined; articles 2–5 distal margin with 1 aesthetasc each, article 5 with 2 additional simple setae distally.

Antenna (Fig. 5D) peduncle 5-articled; article 1 length 0.9 width, glabrous; article 2 length 0.5 article 5 length, with 2 simple setae; article 3 length 0.5 article 5 length, with 1 simple seta and 1 pectinate seta; article 4 length 0.8 article 5 length, with 3 broom setae and 1 simple seta; article 5 length 2.1 width, with 2 robust setae subdistally and 5 broom setae distally; flagellum 2-articled, 0.2 peduncle length, article 1 subequal to article 2 in length, glabrous; article 2 with 5 simple setae distally.

Mandibles, palp 3-articled, incisor process denticulate, molar process spiniform. Left mandible (Fig. 6A) with rounded condyle; *lacinia mobilis* 4-denticulate; palp article 1 with 1 pectinate seta distally; article 2 with 2 pectinate setae distally; article 3 with 7 pectinate setae distally.

Maxillula (Fig. 6B) medial endite with 3 setulate setae; lateral endite with 3 cuspidate and 7 denticulate setae, surface with several cuticular combs.

Maxilla (Fig. 6C) medial endite with 8 setulate and 3 simple setae distally, surface with numerous cuticular combs; both lobes of lateral endite with 4 serrate setae distally (mesial lobe broken).

Maxilliped (Fig. 6D) endite medial margin with 2 coupling hooks, distal margin with 8 pectinate setae, lateral margin with several simple setae, endite surface with 2 setulate setae and several simple setae; palp 4-articled, article 1 distal margin with 1 simple seta; article 2 with 5 setulate setae medially and 3 short setulate setae laterally; article 3 with 3 setulate setae; article 4 with 5 setulate setae and 1 robust seta.

Pereopod 1 (Fig. 6E) shortest and robust, basis longest article, article length ratio (as the relative length of each article to propodus): 2.2: 1.5: 0.9: 0.9: 1.0: 0.3. Basis ventral margin with 5 simple setae; dorsal margin with 1 simple seta proximally and 2 robust setae distally. Ischium ventral margin with 2 simple setae; dorsal margin with 2 robust setae. Merus ventral margin with 3 robust and 2 simple setae; dorsal margin with 2 robust setae and 1 simple seta. Carpus ventral margin with 6 robust setae; dorsal margin with 3 robust setae. Propodus ventral margin with 4 robust and 2 simple setae; distal-most robust seta largest and opposed to dactylus distal claw forming the chela; distal margin with 1 simple seta and 2 robust setae. Dactylus with 1 distal claw, ventral margin with 4 simple setae and 1 short robust seta; dorsal margin with 3 simple setae.

Pereopods 2–7 gradually increasing in length.

Pereopod 2 (Fig. 6F) propodus longest article, article length ratio (as the relative length of each article to propodus): 0.7: 0.5: 0.4: 0.9: 1.0: 0.3. Basis ventral margin with 5 simple setae; dorsal margin with 1 simple seta, 2 robust setae and 1 broom seta. Ischium ventral margin with 4 simple setae; dorsal margin with 3 robust setae. Merus ventral margin with 8 robust and 3 simple setae; dorsal margin with 3 robust setae and 1 simple seta. Carpus ventral margin with 14 robust setae; dorsal margin with 1 broom seta and 8 robust setae. Propodus ventral margin with 5 robust and 5 simple setae; dorsal margin with 7 simple, 1 broom and 4 small pectinate setae. Dactylus distal margin with 1 claw; ventral margin with 1 robust seta and 2 simple setae; dorsal margin with 4 simple setae.

Pereopod 7 (Fig. 6G), propodus longest article, articles length ratio (as the relative length of each article to propodus): 0.9: 0.6: 0.4: 0.9: 1.0: 0.4. Basis ventral margin with 4 simple setae; dorsal margin with 2 simple and 3 robust setae. Ischium ventral margin with 3 simple setae; dorsal margin with 4 robust setae. Merus ventral margin with 6 robust and 2 simple setae; dorsal margin with 2 robust setae and 1 simple seta. Carpus ventral margin with 11 robust setae; dorsal margin with 1 broom seta and 6 robust setae. Propodus ventral margin with 6 robust and 5 simple setae; dorsal margin with 6 simple, 1 broom and 2 small pectinate setae. Dactylus distal margin with 1 claw; ventral margin with 1 robust seta and 2 simple setae; dorsal margin with 4 simple setae.

Operculum (Fig. 7A) length 0.8 at greatest width, narrowing from distal half to rounded apex; distal margin with 55 setulate setae, longest setae at distal end; ventral surface with 10 simple setae (2 missing).

Pleopod 3 (Fig. 7B); protopod medial margin with simple setae; endopod length 0.8 exopod length, with 3 plumose setae distally; exopod 2-articled with many simple setae marginally.

Pleopod 4 (Fig. 7C) uniramous and glabrous.

Pleopod 5 absent.

Uropod (Fig. 7D) cylindrical, length 6.0 at greatest width, with 24 robust setae, distal-most largest, and 3 broom setae.

##### Description of adult male

**(based on paratype MACN-In 45618-b)**. As female except for:

Pleopod 1 (Fig. 7E) with two distal lobes outlined by two furrows; length 1.7 at greatest width; lateral margin with 4 simple setae intercalated between tiny simple setae; distal third with small simple setae sublaterally; lobes distally rounded marginally setulate.

Pleopod 2 (Fig. 7F) protopod subtriangular, length 2.4 at greatest width, margin with 10 simple and 17 setulate setae (some broken), medial-distal margin with several short simple setae; stylet reaches apex of protopod.

##### Remarks.

*Natalianira megalocera* sp. nov. is most similar to *N. bicornuta* sp. nov., both species can be separated from each other by the following features (those of *N. bicornuta* between parentheses): (1) article 1 of antennula extremely large, length/width ratio 3.2 (length/width ratio 1.8), (2) antenna flagellum with 2 articles (flagellum with 1 article), (3) head with two low dorsal bumps (two large cylindrical dorsal protuberances) and, (4) pleotelson lateral margin with 1 large process (2 large processes).

##### Etymology.

The species epithet combines two Greek terms: “mega” meaning large and “kerata” meaning antenna. It refers to the large dorsal process on the first article of the antennula, which resembles the antlers of the Irish elk, *Megaloceros giganteus*.

##### Distribution.

**Figure 9.**
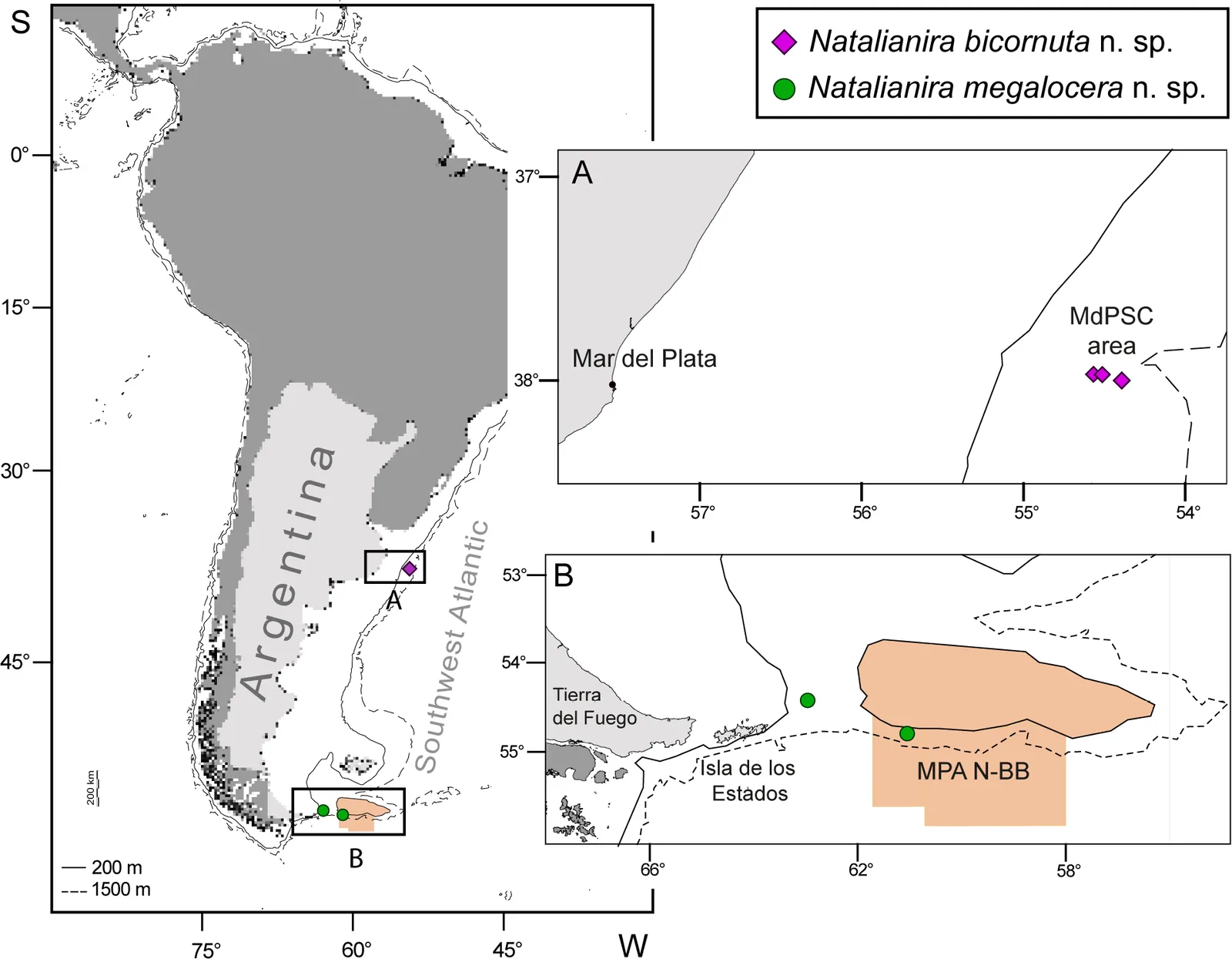
Overview map of the study area. **A, B**. Close-up maps showing the sampling stations with species of *Natalianira*.

This species is only known from off Isla de los Estados and the Marine Protected Area Namuncurá-Burdwood Bank, Argentina, between 647–846 m depth (Fig. 9).

### Identification key to the genera of Katianiridae

**Table:** 

1	Pereonites without dorsal bumps. Pleotelson, lateral margin rounded. Antennula, article 1 short and thin. Pereopod 1, dactylus longer than wide	*** Katianira ***
–	Pereonites with dorsal bumps. Pleotelson, lateral margin with 1 or 2 processes. Antennula, article 1 greatly enlarged. Pereopod 1, dactylus as long as wide	*** Natalianira ***

### Identification key to the species of *Natalianira*

**Table:** 

1	Pereonites with 3 dorsal bumps each; pleotelson with 1 wide lateral process; maxilliped palp of 5 articles	***N. spinosa* Kensley, 1984**
–	Pereonites with 1 dorsal bump each; pleotelson with 1 or 2 narrow lateral processes, maxilliped palp of 4 articles	**2**
2	Head with 2 distinct cylindrical protuberances dorsally, pleotelson with 2 lateral processes; antennula article 1, medial process reaching half-length of article 2; antennal flagellum with 1 article	***N. bicornuta* sp. nov**.
–	Head with 2 low bumps dorsally, pleotelson with 1 lateral process; antennula article 1, medial process greatly produced, extending beyond article 4; antennal flagellum with 2 articles	***N. megalocera* sp. nov**.

## Discussion

Svavarsson (1987) established the family Katianiridae and provided diagnoses of this family and the genera *Katianira* and *Natalianira*. The diagnosis of *Natalianira* has been emended based on the two new species reported herein. The main differences between these two genera can be found in the identification key.

The genus *Natalianira* is exclusively present in the southern hemisphere; in contrast, the genus *Katianira* is predominantly present in the northern hemisphere. The two new species described herein represent the first record of the family Katianiridae from the southwestern Atlantic.

The species of Katianiridae have only been recorded on rare occasions, even in regions where the deep-sea has been relatively well sampled, such as the high latitudes of the northern hemisphere. *Natalianira bicornuta* sp. nov. was obtained in only three out of 29 samples sorted from the MdPSC, whereas *N. megalocera* sp. nov. was obtained in only two out of 12 samples sorted from the MPA N-BB. The occurrence of two new species of the family Katianiridae in a few localities off the coast of Argentina is consistent with the sporadic distribution of members of this family reported by previous authors.

## Supplementary Material

XML Treatment for Natalianira

XML Treatment for Natalianira bicornuta

XML Treatment for Natalianira megalocera

## Additional information

### Artificial Intelligence (AI) use

The authors accept full responsibility for the content of the manuscript, including the disclosure of any use of AI.

No AI tools were used in the preparation of this manuscript.

### Author ORCIDs

S. Calderón López https://orcid.org/0009-0009-7346-2533

D. Roccatagliata https://orcid.org/0000-0002-5718-2875

B. Doti https://orcid.org/0000-0002-5101-829X

### Data availability

All of the data that support the findings of this study are available in the main text.
